# A Prediction Model of Extubation Failure Risk in Preterm Infants

**DOI:** 10.3389/fped.2021.693320

**Published:** 2021-09-22

**Authors:** Zimei Cheng, Ziwei Dong, Qian Zhao, Jingling Zhang, Su Han, Jingxian Gong, Yang Wang

**Affiliations:** Department of Pediatrics, First Affiliated Hospital of Anhui Medical University, Anhui, China

**Keywords:** preterm infant, extubation, mechanical ventilation, early-onset sepsis, hemoglobin

## Abstract

**Objectives:** This study aimed to identify variables and develop a prediction model that could estimate extubation failure (EF) in preterm infants.

**Study Design:** We enrolled 128 neonates as a training cohort and 58 neonates as a validation cohort. They were born between 2015 and 2020, had a gestational age between 25^0/7^ and 29^6/7^ weeks, and had been treated with mechanical ventilation through endotracheal intubation (MVEI) because of acute respiratory distress syndrome. In the training cohort, we performed univariate logistic regression analysis along with stepwise discriminant analysis to identify EF predictors. A monogram based on five predictors was built. The concordance index and calibration plot were used to assess the efficiency of the nomogram in the training and validation cohorts.

**Results:** The results of this study identified a 5-min Apgar score, early-onset sepsis, hemoglobin before extubation, pH before extubation, and caffeine administration as independent risk factors that could be combined for accurate prediction of EF. The EF nomogram was created using these five predictors. The area under the receiver operator characteristic curve was 0.824 (95% confidence interval 0.748–0.900). The concordance index in the training and validation cohorts was 0.824 and 0.797, respectively. The calibration plots showed high coherence between the predicted probability of EF and actual observation.

**Conclusions:** This EF nomogram was a useful model for the precise prediction of EF risk in preterm infants who were between 25^0/7^ and 29^6/7^ weeks' gestational age and treated with MVEI because of acute respiratory distress syndrome.

## Introduction

With the development of perinatology, there has been a rapid increase in the survival rate of premature neonates annually, followed by the increasing incidence of neonatal respiratory distress syndrome (RDS). Although early use of pulmonary surfactant (PS) and non-invasive ventilation [such as continuous positive airway pressure (CPAP), synchronized nasal intermittent positive pressure (SNIPP), bilevel continuous positive distending pressure (BiPAP), etc.] have been proven effective in the prevention and treatment of RDS, some premature neonates with acute RDS still need mechanical ventilation through endotracheal intubation (MVEI). In addition, MVEI is a vital way to save premature neonates after PS and non-invasive ventilation failed. Fifty percent of neonates born at <28 weeks' gestation who received CPAP failed, and 37% of those who received PS failed (1). More than half of neonates born at <29 weeks' gestation required mechanical ventilation (2).

Mechanical ventilation is a double-edged sword, i.e., although it can save the life of neonates, it also might result in adverse outcomes, including ventilator-associated pneumonia (VAP), pneumothorax, pulmonary hemorrhage, and so on (3). If MVEI is done, the physician aims to liberate the neonate from endotracheal intubation as soon as possible to minimize ventilator-associated adverse outcomes. But reintubation frequently occurs in premature neonates in clinical practice. The rate of reintubation in premature neonates ranges from 15.5% to 50% in different studies (1, 4, 5). Reintubation might occur in premature neonates due to lung disease or inadequate respiratory drive, often secondary to increased respiratory work, severe apnea and bradycardia, low oxygen saturation, respiratory acidosis, and severe infection (6, 7). Studies have shown that reintubation might increase the risk of death, VAP, pneumothorax, bronchopulmonary dysplasia (BPD), and neurological damage (1, 7–11).

The timing of extubation is determined by the clinicians and varies according to the clinicians' empirical judgment. Identifying possible predictors of extubation failure (EF)/success (ES) may avoid reintubation and improve clinical outcomes. Studies (1, 11–13) showed greater gestational age, higher 5-min Apgar score, higher pH before extubation, lower pre extubation fraction of inspired oxygen (FiO_2_), lower pre-extubation partial pressure of carbon dioxide (PCO_2_), lower peak FiO2 within the first 24 h of age, non-small for gestational age status, and passing spontaneous breathing trials were associated with a higher likelihood of extubation success (ES). Wang et al. (2) proved that poor acid-base homeostasis 2 h after extubation was associate with EF. Although current predictors of ES, especially spontaneous breathing trials, are highly sensitive to ES, they provide little benefit in the identification of EF (13, 14). Further clinical research is needed to identify objective predictors of EF and improve the clinical outcome of premature neonates with MVEI. Here, we aimed to analyze the demographics, perinatal characteristics, and peri-extubation characteristics of neonates with failed/ successful extubation and to explore the predictors and develop a prediction model that could predict EF.

## Methods

### Study Design

This was a retrospective cohort study approved by the Ethics Committee of the First Affiliated Hospital of Anhui Medical University, Hefei, China.

### Study Participants

Neonates who were born between June 2015 and December 2020 and met the inclusion criteria were recruited from three medical centers, including the First hospital of Anhui Medical University, Second Xiangya Hospital of Central South University, and First Hospital of Jilin University. The neonates recruited from the First Affiliated Hospital of Anhui Medical University born between June 2015 and December 2019 served as the training cohort, and the neonates recruited from all three medical centers born between January 2020 and December 2020 served as the validation cohort. The inclusion criteria were as follows: (1) 25^0/7^ to 29^6/7^ weeks' gestational age; (2) born in the obstetrics department at the same hospital with NICU, transferred to NICU immediately after birth and treated with MVEI within the first 24 h of postnatal age (to reduce the heterogeneity of the study population). The exclusion criteria were as follows: (1) died before extubation; (2) accidental extubation; (3) received surgical treatment within 5 days after extubation; (4) congenital malformation of vital organs or genetic/metabolic diseases; (5) the lack of important clinical data.

### Data Collection

We collected clinical characteristics (demographics, perinatal characteristics, and peri-extubation characteristics) and clinical outcomes of the infants from the medical records. Demographics were collected, including gender, gestational age (GA), birth weight (BW), small for gestational age (SGA), assisted reproduction, cesarean delivery, multiple births, and maternal age at pregnancy. Perinatal characteristics were collected, including hypertensive disorder complicating pregnancy of the mother, gestational diabetes mellitus of mother, antenatal steroids, prolonged rupture of the membrane [>18 h], 1-min Apgar score, 5-min Apgar score, intubated in the delivery room, epinephrine in the delivery room, PS administration, hypoglycemia, arterial blood gases [pH, arterial partial pressure of carbon dioxide (PaCO_2_)] within 0.5 h before intubation, non-invasive ventilation before intubation, age of starting enteral feeding, and early-onset sepsis (EOS). Peri-extubation characteristics were collected, including ventilator mode at extubation [high-frequency ventilation (HFV), conventional mechanical ventilation (CMV)], hematology examination [hemoglobin concentration (HB), serum albumin (ALB), serum globulin (GLB)] within 48 h before extubation, arterial blood gases within 0.5 h before extubation (pH, PaCO_2_), highest fraction of inspired oxygen (FiO_2_) after intubation, FiO_2_ at extubation, first MVEI duration, weight at extubation, postmenstrual age (PMA) at extubation, and caffeine treatment. Clinical outcomes were collected, including death, BPD, neonatal necrotizing enterocolitis (NEC), late-onset sepsis (LOS), VAP, pulmonary hemorrhage, pneumothorax, retinopathy of prematurity (ROP), age at total enteral feeding, length of time on MVEI, length of time on hospital stay, length of time on oxygen, antibiotic utilization rate, daily weight gain, and daily hospitalization cost.

### Definitions

EF was defined as the need for reintubation within 5 days after extubation. ES was defined as survival for more than 5 days without MVEI. Intubation/reintubation and extubation criteria were based on the “The Neonatal Mechanical Ventilation Routine” (15) that was published in May 2015. The decisions regarding initial endotracheal intubation, the timing of extubation, post-extubation support, and the need for reintubation were at the discretion of the chief physician. The neonate could be intubated if they met any of the following criteria: (1) frequent apneas, medication or non-invasive ventilator intervention was ineffective; (2) neonates with RDS required PS treatment; (3) FiO_2_ > 0.6–0.7, arterial partial pressure of oxygen <50–60 mmHg or transdermal oxygen saturation <85%; (4) PaCO_2_ > 60–65 mmHg, with persistent acidosis (pH <7.20). The reintubation criteria were the same as those for initial intubation. If an infant using CMV met all of the following criteria, then extubation was done: (1) the primary disease of the neonate improved, the infection was controlled, and the general condition (including stable breathing, stable oxygen saturation, and active response) was good; (2) Peak inspiratory pressure ≤ 18 cm H_2_O, positive end-expiratory pressure at 3–6 cm H_2_O, breathing rate at 12–20 breaths/min, FiO_2_ ≤ 0.4. There was no uniform weaning standard for HFV. Before weaning from HFV, FiO_2_ was lowered, then MAP was lowered, and finally, amplitude was adjusted according to the PaCO_2_. If HFV was being used, it was usually transferred to CMV and then weaned. In this study, non-invasive ventilator was either continued after extubation, or withdrawn directly.

Hypoglycemia was defined as peripheral blood glucose <40 mg/dL after birth. Sepsis was defined as systemic inflammatory reaction syndrome caused by various pathogens, including culture-positive and culture-negative septicemia. Definition of culture-negative sepsis refers to culture-negative, a clinical manifestation of a systemic infection, antibiotics ≥3 days, laboratory findings (WBC < 5 × 10^9^/mL or ≥ 30 × 10^9^/L, CRP> 10 mg/L). Early-onset sepsis refers to sepsis that occurs within 3 days after birth; otherwise, it was known as late-onset sepsis. In this study, caffeine treatment implied the use of caffeine citrate before the first extubation and was maintained for over 2 more days after extubation. The method of administration was the first loading dose of 20 mg/kg, and the maintenance dose of 5 mg/kg·d. BPD was defined for infants treated with > 21% oxygen for ≥28 days and/or at 36 weeks postmenstrual age. The diagnosis of pneumothorax is confirmed by a bedside chest radiograph. VAP refers to pneumonia that occurs when infants receive mechanical ventilation for more than 48 h. NEC is defined as stage II and III of correction Bell's staging system (16). ROP was defined as any Stage 3 ROP with plus disease, and Stages 4 or 5 of ROP (17).

### Statistical Analysis

The preterm infants in the training cohort were divided into EF groups and ES groups according to whether to reintubate within 5 days after extubation. For the infants who failed extubation, Kaplan-Meier survival curves were plotted to explore the timing of EF. Univariate logistic regression analysis was performed to identify the risk factors for EF. All *P*-values were two-sided and *P* < 0.05 were considered significant. Among all factors that were confirmed to be statistically significant, such as antenatal steroids, 5-min Apgar score, EOS, hemoglobin concentration before extubation, pH before extubation, PCO_2_ before extubation, and caffeine treatment before and after extubation, stepwise discriminant analysis [method: Wilk's lambda; criteria: use probability of F (entry of 0.1 and removal of 0.15)] was performed to select the useful combination of factors that could precisely predict EF. A nomogram for EF was created based on the multivariate logistic regression model using the selected factors. Receiver operator characteristic (ROC) curve was performed to evaluate the predictive value of the model. Next, we carried out Harrell's concordance index (C-index) and calibration plot in both training and validation cohorts to assess the performance of the model. A concordance index is a numerical measure of discriminative ability and calibration plots are graphic evaluations of predictive ability that compare observed probabilities with nomogram-predicted probabilities (18). Finally, we compared the clinical outcomes of the preterm infants in both the groups. We used the χ^2^ test or Fisher's exact test to compare the categorical variables, and Mann-Whitney *U*-test to compare the continuous variables. Statistical analysis was conducted using the IBM SPSS Statistics (version 25.0; SPSS Inc., Chicago, IL) and the R software (Version 4.0.3; http://www.R-project.org).

## Results

### Demographics of Infants

We eventually enrolled 128 infants as training cohort. Of these 128 infants (Table 1), they had a median GA of 28.29 weeks with IQR of 27.43–29.29 weeks, had a median BW of 1,060 grams with an IQR of 900–1,250 grams. Seventy-three (60.15%) infants were males, 18 (14.06%) were SGA, 44 (34.38%) were assisted reproduction, 64 (50.00%) were cesarean delivery, 40 (31.25%) were multiple births. The median and IQR of maternal age was 27 (29, 32) years.

**Table 1:** Characteristics of 128 preterm infants and Univariate analysis of risk factors for extubation failure.

**Characteristics**		**Univariate regression analysis**	
		**Odds ratio (95% C.I.)**	***P*-value**
**Demographics**			
Male sex, no. (%)	73 (60.15)	1.38 (0.62, 3.11)	0.432
GA, week, median (IQR)	28.29 (27.43, 29.29)	0.83 (0.60, 1.13)	0.234
BW, g, median (IQR)	1,060 (900, 1250)	1.00 (0.99, 1.01)	0.993
SGA, no. (%)	18 (14.06)	0.49 (0.13, 1.80)	0.281
Assisted reproduction, no. (%)	44 (34.38)	1.40 (0.63, 3.13)	0.412
Cesarean delivery, no. (%)	64 (50.00)	0.57 (0.26, 1.26)	0.167
Multiple births, no. (%)	40 (31.25)	1.44 (0.64, 3.28)	0.379
Maternal age, year, median (IQR)	27 (29, 32)	0.98 (0.91, 1.06)	0.584
**Perinatal characteristics**			
Hypertensive disorder complicating pregnancy, no. (%)	36 (28.125)	0.43 (0.16, 1.16)	0.096
Gestational diabetes mellitus, no. (%)	22 (17.19)	1.00 (0.36, 2.79)	0.993
Antenatal steroids, no. (%)	98 (76.56)	0.30 (0.16, 0.90)	0.028^*^
Premature rupture of membranes (>18 h), no. (%)	43 (33.59)	0.60 (0.25, 1.43)	0.250
1-min Apgar score, median (IQR)	5 (3, 6)	0.84 (0.69, 1.02)	0.078
5-min Apgar score, median (IQR)	7 (6, 8)	0.70 (0.54, 0.90)	0.006^*^
Intubated in the delivery room, no. (%)	81 (63.28)	1.16 (0.51, 2.61)	0.726
Epinephrine in the delivery room, no. (%)	11 (8.59)	1.00 (0.25, 3.99)	0.996
Pulmonary surfactant treatment, no. (%)	125 (97.66)	–	0.999
Hypoglycemia, no. (%)	12 (9.38)	1.37 (0.39, 4.88)	0.626
Arterial blood gases before intubation:			
pH_1, median (IQR)	7.31 (7.25, 7.37)	0.21 (0.00, 15.66)	0.475
PCO_2__1, mmHg, median (IQR)	42.25 (34.33, 48.55)	1.00 (0.97, 1.04)	0.837
Noninvasive ventilation before intubation, no. (%)	8 (6.25)	1.65 (0.37, 7.30)	0.509
Age of starting enteral feeding, day, median (IQR)	2.74 (1.93, 4.00)	1.16 (0.98, 1.38)	0.083
EOS, no. (%)	38 (29.69)	2.72 (1.20, 6.17)	0.017^*^
**Peri-extubation characteristics**			
Ventilator mode at extubation:			
HFV, no. (%)	5 (3.91)	1.82 (0.29, 11.37)	0.523
CMV, no. (%)	123 (96.09)	0.55 (0.09, 3.44)	0.523
Hematology examination before extubation:			
HB, g/L, median (IQR)	127 (113, 147)	0.98 (0.96, 1.00)	0.017^*^
ALB, g/L, median (IQR)	29.95 (27.00, 32.65)	0.96 (0.88, 1.06)	0.421
GLB, g/L, median (IQR)	15.8 (12.1, 19.7)	1.02 (0.96, 1.09)	0.491
Arterial blood gases before extubation:			
pH_2, median (IQR)	7.31 (7.25, 7.37)	0.00 (0.00, 0.15)	0.006^*^
PCO_2__2, mmHg, median (IQR)	41.67 (34.90, 51.25)	1.04 (1.01, 1.07)	0.009^*^
Highest FiO_2_ after intubation, %, median (IQR)	40 (30, 49)	1.02 (0.99, 1.05)	0.307
FiO_2_ at extubation, %, median (IQR)	25 (21, 25)	1.07 (0.98, 1.18)	0.135
First MVEI duration, day, median (IQR)	5.48 (3.05, 10.33)	1.02 (0.97, 1.08)	0.423
Weight at extubation, g, median (IQR)	1,083 (950, 1,210)	1.00 (1.00, 1.002)	0.834
PMA at extubation, week, median (IQR)	29.36 (28.57, 30.25)	0.94 (0.70, 1.26)	0.677
Caffeine treatment, no. (%)	79 (61.72)	0.24 (0.11, 0.55)	<0.001^*^

* *P < 0.05*.

*GA, gestational age; BW, birth weight; SGA, small for gestational age; PCO_2_, partial pressure of carbon dioxide; EOS, early onset sepsis; HFV, High-frequency ventilation; CMV, conventional mechanical ventilation; HB, hemoglobin concentration; ALB, serum albumin; GLB, serum globulin; FiO_2_, fraction of inspired oxygen; MVEI, mechanical ventilation through endotracheal intubation; PMA, postmenstrual age*.

### Timing and Cause of EF

**Figure 1:**
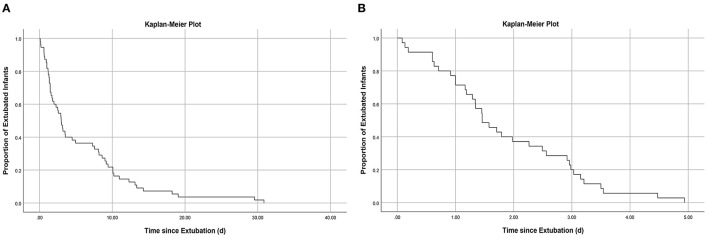
**(A)** Kaplan–Meier curve showing proportion of infants remaining extubated during hospitalization: Among those who failed extubation during hospitalization, 63.6% underwent reintubation within 5 days; **(B)** Kaplan–Meier curve showing proportion of infants remaining extubated within 5 days: Among those who failed extubation within 5 days, 80.0% underwent reintubation within 3 days.

In the training cohort, 55/128 (43.0%) of the infants who received MVEI treatment were reintubated during hospitalization, and 35/55 (63.6%) were reintubated within 5 days after extubation (Figure 1A), the median reintubation time was 2.98 days with an IQR of 1.34–9.17 days. Based on whether reintubation was required within 5 days after extubation, all infants of the training cohort were divided into the EF group (*n* = 35) and the ES group (*n* = 93). In the EF group, 28/35 (80%) infants were reintubated within 3 days (Figure 1B), and the median reintubation was 1.46 days with an IQR of 1.00–2.96 days. The cause of EF: 12/35 (34.29%) had significant apnea and bradycardia, 23/35 (65.71%) had CO_2_ retention or/and hypoxemia including severe infection 11/35 (31.43%), atelectasis 7/35 (20.00%), and pulmonary hemorrhage 5/35 (14.29%).

### Factors Associated With EF

In clinical practice, ventilator mode at extubation and the administration of PS might affect the occurrence of EF. In this study, 96.09% of infants were weaned with CMV, 3.91% were weaned with HFV. Among those who were weaned with HFV, 2 were in the EF group and 3 were in the ES group, there was no statistical significance between the two groups (*P* = 0.614, Fisher's exact test). The rate of PS administration in this study was 97.66%. Further analysis of the data showed that 35 infants in the EF group used PS and 90 infants in the ES group, there was no statistical significance between the two groups (*P* = 0.561, Fisher's exact test).

**Figure 2:**
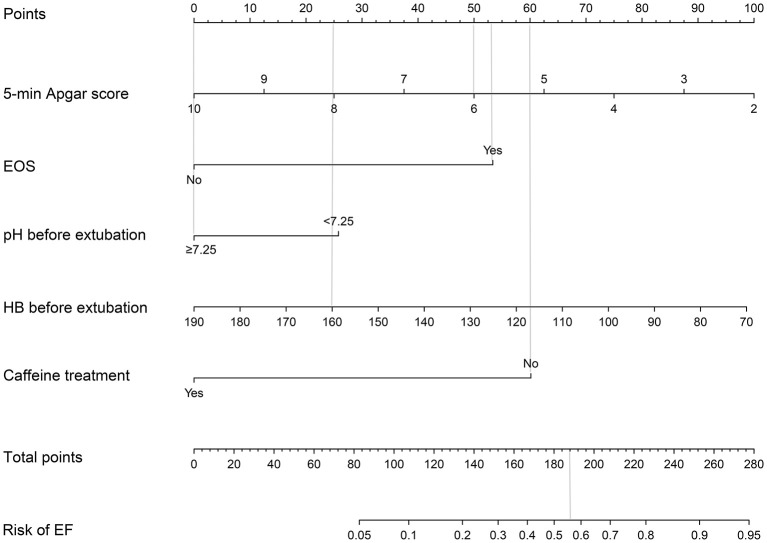
Extubation failure risk nomogram. The extubation failure risk nomogram was developed in the cohort, with 5-min Apgar score, the dignosis of EOS, pH before extubation, HB before extubation, and the caffeine treatment. To estimate the probability of extubation failure, mark infant values at each axis, draw a straight line perpendicular to the point axis, and sum the points for all variables. Next, mark the sum on the total point axis and draw a straight line perpendicular to the probability axis. EOS, early-onset sepsis; HB, hemoglobin concentration; EF, extubation failure.

Table 1 presents the clinical characteristics of infants, including perinatal and peri-extubation characteristics in the two groups. In univariate logistic regression analysis, antenatal steroids (95% CI: 0.16–0.90), 5-min Apgar score (95% CI: 0.54–0.90), EOS (95% CI: 1.20–6.17), hemoglobin concentration before extubation (95% CI: 0.96–1.00), pH before extubation (95% CI: 0.00–0.15), PCO_2_ before extubation (95%CI: 1.01–1.07), and caffeine treatment (95% CI: 0.11–0.55) were identified as the risk factors of EF. We selected 5-min Apgar score, EOS, pH before extubation, hemoglobin concentration before extubation, and caffeine treatment as the best combination of factors to predict EF through stepwise discriminant method. A nomogram for EF was constructed based on the multivariate logistic regression model using these selected factors (Table 2; Figure 2).

**Table 2:** Prediction factors for extubation failure in MVEI infants.

**Intercept and variable**	**Prediction model**		
	**β**	**Odds ratio (95% C.I.)**	***P*-value**
5-min Apgar score^a^	−0.376	0.687 (0.516, 0.913)	0.010^*^
EOS^b^	1.792	6.002 (2.063, 17.465)	<0.001^*^
pH before extubation^c^	−6.093	0.002 (0.000, 0.521)	0.028^*^
HB before extubation^d^	−0.022	0.978 (0.957, 0.999)	0.042^*^
Caffeine treatment^e^	−1.800	0.165 (0.060, 0.455)	<0.001^*^

* *P < 0.05. β is the regression coefficient; C.I., confidence interval; EOS, early onset sepsis; HB, hemoglobin concentration*.

a *Odds ratio represents decreased odds per increase in 5-min Apgar score of 1*.

b *Odds ratio represents increased odds if a neonate was diagnosed as EOS*.

c *Odds ratio represents decreased odds per increase in pH by 1*.

d *Odds ratio represents decreased odds per increase in hemoglobin concentration before extubation by 1*.

e *Odds ratio represents decreased odds if a neonate was treated with caffeine*.

### Clinical Outcomes of Two Groups

Compared with the outcomes of the two groups, infants in the EF group had a higher rate of mortality (14.3 vs. 3.2%), higher rate of VAP (51.4 vs. 20.4%), higher rate of pulmonary hemorrhage (37.1 vs. 18.3%), longer time on MVEI among survivors [median (IQR), 16.83 (8.65, 26.45) vs. 6.74 (3.32, 12.96)], higher antibiotic administration rate [median (IQR), 0.56 (0.45, 0.77) vs. 0.46 (0.32, 0.61)], slower weight gain [median (IQR), 10.11 (6.39, 12.81) vs. 12.72 (11.03, 14.57)], and higher hospitalization costs [median (IQR), 1,533.14 (1,364.65, 1,660.43) vs. 1,395.16 (1,242.83, 1,551.66)] (Table 3). The above indicators were statistically significant (*P* < 0.05).

**Table 3:** Outcomes for 128 preterm infants who had extubation failure vs. extubation success.

**Outcomes**	**Extubation failure (*n* = 35)**	**Extubation success (*n* = 93)**	***P*-value**
Death before discharge, no. (%)	5 (14.3)	3 (3.2)	0.035^*^
BPD among survivors^¶^, no. (%)	19 (63.3)	51 (56.7)	0.521
BPD or death, no. (%)	24 (68.6)	54 (58.1)	0.277
NEC (≥ stage 2), no. (%)	7 (20.0)	8 (8.6)	0.074
LOS, no. (%)	9 (25.7)	36 (38.7)	0.17
VAP, no. (%)	18 (51.4)	19 (20.4)	<0.001^*^
Pneumorrhagia, no. (%)	13 (37.1)	17 (18.3)	0.025^*^
Pneumothorax, no. (%)	1 (2.9)	2 (2.2)	0.814
ROP among survivors^¶^ (≥ stage 3), no. (%)	6 (20.0)	10 (11.1)	0.226
Age at total enteral feeding among survivors^¶^, d, median (IQR)	46.50 (28.75, 57.75)	48.00 (38.75, 61.50)	0.350
Length of time on MVEI among survivors^¶^, d, median (IQR)	16.83 (8.65, 26.45)	6.74 (3.32, 12.96)	<0.001^*^
Length of time on hospital stay among survivors^¶^, d, median (IQR)	68 (57.1, 77.25)	65.5 (53, 75.25)	0.272
Length of time on oxygen among survivors^¶^, d, median (IQR)	37 (32.5, 52)	36.5 (21, 52)	0.244
Antibiotic utilization rate, median (IQR)	0.56 (0.45, 0.77)	0.46 (0.32, 0.61)	0.004^*^
Daily weight gain, gram, median (IQR)	10.11 (6.39, 12.81)	12.72 (11.03, 14.57)	0.002^*^
Daily hospitalization cost, yuan/day, median (IQR)	1,533.14 (1,364.65, 1,660.43)	1,395.16 (1,242.83, 1,551.66)	0.009^*^

*Antibiotic utilization rate: Duration of antibiotic use/length of hospital stay*;

¶ *excludes 8 infants who died before hospital discharge*;

* *P < 0.05*;

*BPD, bronchopulmonary dysplasia; NEC, neonatal necrotizing enterocolitis; LOS, late-onset sepsis; VAP, ventilator-associated pneumonia; ROP, retinopathy of prematurity; MVEI, mechanical ventilation through endotracheal intubation*.

### Prediction Model for EF

**Figure 3:**
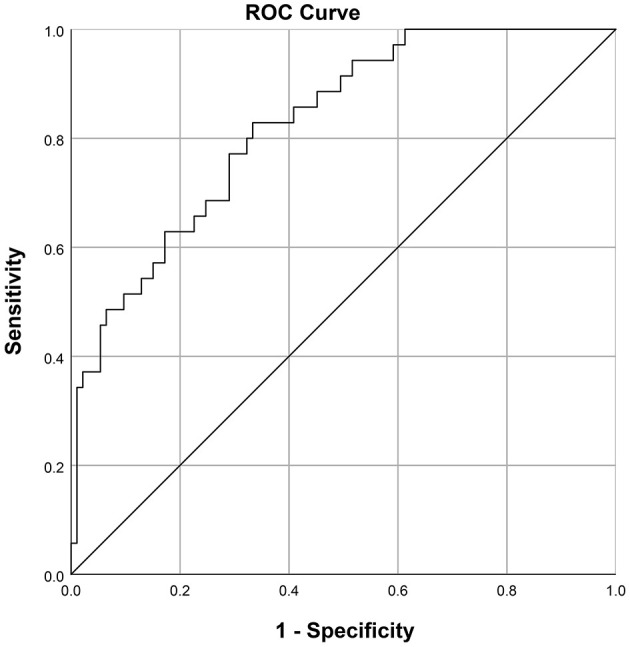
Receiver operating characteristic curve for the prediction model: Area under the curve was 0.824 (95% confidence interval 0.748–0.900).

The nomogram which could predict the probability of EF was created using the factors of 5-min Apgar score, EOS, pH before extubation, hemoglobin concentration before extubation, and caffeine treatment (Figure 2). The area under the ROC curve was 0.824 (95% CI: 0.748–0.900; Figure 3). The model with highest Youden index defined EF risk as 0.207, the sensitivity is 0.829, the specificity is 0.667, the positive predictive value is 0.483, and the negative predictive value is 0.912.

The usage of the predictive model was drawn with an assumptive preterm infant with a 5-min Apgar score of 6, EOS, HB before extubation of 160 g/L, pH before extubation of 7.35, and no caffeine treatment. The points for 5-min Apgar score, EOS, HB before extubation, pH before extubation, and caffeine treatment were 50, 53, 25, 0, and 60, respectively. The total point added up to 188 for this infant, which represented ~0.56 of the probability of EF.

**Figure 4:**
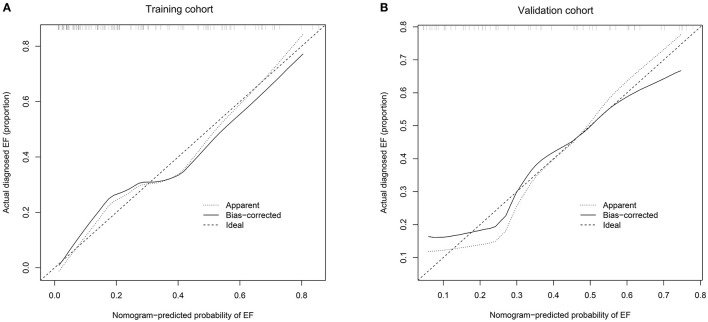
**(A)** Calibration curves of the nomogram to predict extubation failure in training cohort; **(B)** Calibration curves of the nomogram to predict extubation failure in validation cohort. The horizontal axis shows the predicted probability of extubation failure, and the vertical axis shows the observed probability of extubation failure. Perfect prediction would correspond to the 45° broken line. The dotted and solid lines indicate the observed (apparent) nomogram performance before and after bootstrapping. EF, extubation failure.

The performance of this nomogram was assessed by C-index and calibration plots. Next, we collected 58 preterm infants who met the inclusion and exclusion criteria as a validation cohort to validate our model. The C-index in the training and validation cohorts was 0.824 and 0.797, indicating a high discrimination of the nomogram. The calibration plots also showed high coherence between the predicted probability of EF and actual observation (Figure 4), which indicated good calibration of the model.

## Discussion

Infants with EF are known to be more likely to have a higher rate of mortality and morbidity, including VAP, pneumothorax, BPD, neurological damage, etc. Currently, the decision to extubate is based on clinicians' discretion and is influenced by clinical evaluation, blood gas variables, oxygen requirement, and level of ventilator support. There is a lack of strong evidence to support the use of any predictor of extubation readiness in preterm infants over clinical judgment alone. Although current studies on spontaneous breathing trials are highly sensitive to ES, they provide little benefit in the identification of EF (13, 14). Notably, studies concentrating on predicting the EF in preterm infants account for less of the studies related to ventilators.

The existing studies have shown a high rate of EF in preterm infants. Chawla et al. (1) (*n* = 1,071, <28 weeks GA) showed that the rate of EF within 5 days in preterm infants was 37–50%. Chen et al. (4) (*n* = 206, <37 weeks GA) suggested that the rate EF within 7 days was 15.5–33.9%. Wissam et al. (19) (*n* = 194, ≤ 1,250 grams BW) showed that the cumulative rate of EF was high within 3–7 days after extubation. In our study, we found that 55/128 (43.0%) neonates with MVEI were reintubated during hospitalization, and 35/128 (27.3%) were reintubated within 5 days after extubation. More than half (35/55) neonates were reintubated within 5 days of extubation, which was consistent with the results of Wissam et al. A study of Wang et al. (2) showed the reasons for reintubation within 7 days after extubation included significant apnea and bradycardia, pulmonary hemorrhage, respiratory acidosis, pneumothorax, lung collapse, and increased work of breathing; cases who were reintubated >7 days after extubation were due to infection-associated episodes (culture-proved sepsis, pneumonia, NEC, etc.). In this study, we defined EF as needing reintubation within 5 days after extubation to include more neonates with EF and to avoid new disease becoming the cause of reintubation.

Similar to the previous studies (1, 12), a lower 5-min Apgar score, lower pH before extubation were associated with a higher likelihood of EF, which implied that infants were more likely to need reintubation. The lower 5-min Apgar score indicated that the hypoxia period might be longer (might last for several min or longer, worse than the lower 1-min Apgar score) in a neonate's early life. Also, the neonate's lung and brain might suffer from serious hypoxia injury, which may affect the recovery of respiratory function. Blood gas pH before extubation is an important marker for extubation readiness. The lower pH indicates that the current oxygen exchange capacity of the lung cannot meet the body's demand for oxygen supply.

Our study found that treatment with caffeine prior to extubation and maintained for more than 2 days after extubation reduced the risk of EF compared with non-use of caffeine. Caffeine administration can quickly and effectively increase the sensitivity of the respiratory tract to carbon dioxide, reduce the frequency of apneas, increase the stroke volume of the left ventricle, increase the minute ventilation and tidal volume, and improve lung function, thereby improving the blood oxygen partial pressure in the arteries and reducing the failure rate of weaning (20). The use of caffeine before and after extubation can reduce the occurrence of recurrent respiration and hypoxemia, improve the respiratory function of premature neonates, increase the pH value and decrease the PaCO_2_, and reduce the incidence of apnea and reintubation (21). Amaro et al. (22) noted that caffeine might increase the rate of mortality in neonates, but the reliability of the results was challenged due to the small sample size and the absence of statistical significance. A meta-analysis (23) of six randomized controlled trials, including 620 neonates, suggested that high-dose caffeine could reduce the risk of death. Although studies have shown that the use of caffeine might be beneficial for the long-term prognosis of preterm infants, the timing of caffeine administration is still controversial (22, 24). Our analysis results proved that the rate of EF was lower if the caffeine is maintained for over 2 more days after extubation. However, it has failed to give an optimal maintenance time to minimize the rate of EF, and further research is needed to explore it in the future.

Our study showed that EOS was associated with EF. Similar to our research, Capasso et al. (25) found that LOS was related to failure of non-invasive support. EOS and LOS have differences in high-risk factors and pathogenic bacteria. In our study, EF occurred mostly after the diagnosis of EOS. Therefore, our research suggests that EOS may play a role in facilitating EF. However, the study of Capasso et al. did not specify the time sequence of LOS and failure of non-invasive support. It is possible that EF increases LOS, or that LOS causes EF, or the two influence each other. We speculated that the relationship between EOS and EF might have the following mechanism. First, sepsis might manifest as dyspnea, apnea, cyanosis, etc., among which apnea or respiratory distress could be the starting manifestation of EOS (26). For neonates who were extubated within 3 days of birth, they might be reintubated due to sepsis. Second, inflammatory factors can attack immature lung tissues during inflammatory storms (27, 28). Once the alveolar cell and lung interstitial tissue were damaged by inflammation, the ventilation function and pulmonary vascular hemodynamics were affected, and this damage might be irreversible. Third, sepsis might be complicated by encephalitis, leading to dysfunction of the respiratory center.

EOS in our study included culture-positive and culture-negative septicemia. Many neonates are diagnosed with a “probable or possible” sepsis or a “presumed symptomatic infection but no bacterial cause identified”; conditions often referred to as “culture-negative sepsis” (29). Robust epidemiological data on culture-negative sepsis are limited, especially in younger gestational age preterm infants. The incidence of EOS is substantially higher in preterm infants (30). A study in Norway showed that the incidence of negative blood culture and culture-proven EOS in term newborns was 15.12% (31). The incidence of EOS in our study was 29.7% (38/128). The infants in our study have two high-risk factors: very preterm or extremely preterm (gestational age of 25^0/7^~29^6/7^ weeks), invasive procedures (100% with endotracheal intubation; most with an indwelling nasogastric tube, umbilical vein catheter, or peripheral venous catheter). Thus, the higher incidence of EOS seems reasonable. This study provides data on the incidence of EOS, including culture-positive and culture-negative septicemia, on the condition that epidemiological data are limited.

Our study showed that the lower HB within 48 h before extubation was associated with EF, which was not mentioned in the published literature that we can retrieve. The result suggests that if infants with invasive ventilation have anemia, indications for red blood cells transfusion might need to be appropriately broadened. As we know, hemoglobin is responsible for transporting oxygen and carbon dioxide as the important component of red blood cells. Previous studies have reported a significant association between anemia and respiratory disease. Watanabe et al. (32) showed that hemoglobin concentration was probably related to brain development. Duan et al. (33) demonstrated that early anemia (≤14 days of postnatal life) was associated with an increased risk of BPD in preterm infants. Hussain et al. (34) reported that low HB levels were a risk factor for acute lower respiratory tract infections. In traditional physiology, anemia is associated with an increase in heart rate and cardiac output. We speculate that low HB before extubation was associated with EF because of its influence on the respiratory center, lung, and hemodynamics. Lower HB might decrease oxygen delivery to the respiratory center of the brain and cause respiratory symptoms, including tachypnea, dyspnea, and apnea, which may increase days of mechanical ventilation. Low HB levels reduce the ability of blood to transport oxygen from the lungs to the tissues, leading to increased anaerobic glycolysis and increased production of lactic acid, resulting in a compensatory increase in respiratory rate and heart rate, and an increased burden on the heart and lungs.

Our results were not consistent with the study by Manley et al. (11) on extremely premature neonates (n = 174, <28 weeks GA), which found that higher GA was associated with ES. In univariate regression analysis, the 95% CI of gestational age was 0.60–1.13. This was probably due to the composition of the research population. In China, the termination of pregnancy with a fetal gestational age of fewer than 28 weeks is defined as abortion in obstetrics. It often happens that fetuses with lower gestational age or weaker viability after birth are more likely to be aborted or be abandoned treatment by their parents. Therefore, the smaller the gestational age of a fetus was, the less likelihood of the fetus being transferred to our NICU and received MVEI. The relationship between GA and EF needs to be further confirmed.

The primary limitation of this study is that the included predictors were not comprehensive. Researches by Ding et al. (35) and Capasso et al. (25) showed that different non-invasive ventilation modes (such as SNIPP, CPAP, BiPAP, etc.) after extubation had different reintubation rates. However, due to the limited study conditions, the non-invasive ventilation modes were not included in our analysis. Future studies can conduct randomized controlled trials to further explore the relationship between different non-invasive ventilation modes and EF. Intraventricular hemorrhage (IVH) and patent ductus arteriosus (PDA) are known to occur more frequently in preterm infants (36, 37). Thus, the damage of IVH to nerve function and the influence of PDA on hemodynamics might also be risk factors for EF. Our NICU did not routinely perform bedside echocardiographic and intracranial ultrasound until October 2017. Only infants with high suspicion of heart or brain problems would qualify for bedside ultrasound by the time. Thus, there was lack of data on cardiac and cranial imaging in the medical records of some infants before the first extubation. Considering the accuracy of the study, we did not include PDA and IVH in the analysis. Future studies could focus on the relationship between IVH, PDA and EF. The secondary limitation of this study is the small sample size due to the following reasons: (1) The number of preterm infants is small, especially lower gestational age preterm infants. And extremely premature infants were usually abandoned treatment; (2) The INSURE approach is the best choice for most premature infants with mild RDS and reduces the need of MVEI in some severe RDS infants; (3) Strict inclusion criteria were set to reduce the heterogeneity of the study population. Multi-center prospective and larger sample size of studies should be performed to verify the finding and find more predictors.

The high C-index and the good coherence between the predicted probability and actual observation of the model indicate that the modeling has better performance. The model with highest Youden index defined EF risk as 0.207 and predicted extubation failure with a sensitivity of 0.83 and a specificity of 0.67. The study of Shalish et al. (13) shows that spontaneous breathing trials with highest Youden index predicted extubation success with a sensitivity of 0.93 and a specificity of 0.39. The two can complement each other as extubation preparations. The strengths, limitations and improvement directions of our model are listed as follows. The main strengths: (1) The nomogram can rely on a user-friendly digital interface to assist doctors in making clinical decisions. And this is the first study that used a nomogram to predict EF in preterm infants, which was demonstrated good discrimination and calibration power in the validation; (2) The model could facilitate the individualized prediction of EF using five available variables. Clinicians could combine the infant's individual condition with available predictors to prepare for extubation. The main limitations: (1) Our cohort was not representative of all neonates with MVEI. Different countries might have different intubation/reintubation and extubation practices that limit the generalizability of the model; (2) Though we have tried to make inclusion criteria and data collection more rigorous, undiscovered bias and better predictors might exist since it is a retrospective study. Improvement directions: (1) The model established in this study is the prediction model of all-cause EF. Establishing predictive models, respectively, based on the causes of EF will make it possible to predict the EF and its causes, which is conducive to take targeted preventive measures for infants at high risk of EF. (2) Discovering and incorporating more and better predictors in the model.

Our study noted that neonates who failed extubation had higher rates of mortality, VAP, pulmonary hemorrhage, and other negative outcomes among survivors, including longer time on MVEI, higher antibiotic administration rate, and higher hospitalization costs. These findings are consistent with published researches that suggest a relatively poor prognosis for EF.

## Conclusions

Our study suggested that a lower 5-min Apgar score, EOS, lower pH before extubation, lower HB before extubation, and non-use of caffeine treatment before and after extubation were the key factors that determined EF for infants treated with MVEI. Based on our research, we conclude that the following measures might reduce the rate of EF for a preterm infant with a gestational age of 25^0/7^~29^6/7^ weeks: (1) In the delivery room, the best resuscitation teams and facilities should be ensured to improve the 5-min Apgar score; (2) After transferring to the NICU, it is important to take measures to prevent sepsis, especially for neonates with a low 5-min Apgar score; (3) Reduce unnecessary venous blood collection. When a neonate passes the spontaneous breathing trial, clinicians should monitor the neonate's HB level before weaning. If the 5-min Apgar score is found to be low or sepsis has been diagnosed, and the hemoglobin is at a relatively low level, indications for red blood cells transfusion could be appropriately broadened; (4) Extubation at pH <7.25 is not recommended; (5) It is recommended to administer caffeine before extubation and maintain it for over 2 days after extubation.

Our study provides a reference basis for clinicians to choose the timing of extubation and improves the situation that extubation relies on the subjective judgment of clinicians in some way. Our cohort was not representative of all preterm infants with invasive mechanical ventilation; thus, qualified medical centers could try to organize a multi-center prospective study to verify the conclusion and explore better models.

## Data Availability Statement

The raw data supporting the conclusions of this article will be made available by the authors, without undue reservation.

## Ethics Statement

The studies involving human participants were reviewed and approved by Ethics Committee of the First Affiliated Hospital of Anhui Medical University. Written informed consent from the participants' legal guardian/next of kin was not required to participate in this study in accordance with the national legislation and the institutional requirements.

## Author Contributions

ZC, ZD, and QZ: conception and study design, collection, analysis, and interpretation of data, and writing the first draft of the manuscript. JZ, SH, and JG: collection, analysis, and interpretation of data and drafting the manuscript for important intellectual content. YW: conception and study design, drafting the manuscript for important intellectual content, review and editing of the manuscript, and the decision to submit the paper for publication. All authors contributed to the article and approved the submitted version.

## Funding

This work was supported by the Public Welfare Technology Application Research Linkage Project of Anhui Provincial Science and Technology Department (grant number 1704f0804018).

## Conflict of Interest

The authors declare that the research was conducted in the absence of any commercial or financial relationships that could be construed as a potential conflict of interest.

## Publisher's Note

All claims expressed in this article are solely those of the authors and do not necessarily represent those of their affiliated organizations, or those of the publisher, the editors and the reviewers. Any product that may be evaluated in this article, or claim that may be made by its manufacturer, is not guaranteed or endorsed by the publisher.

## Abbreviations

RDS: Respiratory distress syndrome
PS: Pulmonary surfactant
CPAP: Continuous positive airway pressure
SNIPP: Synchronized nasal intermittent positive pressure
BiPAP: Bilevel continuous positive distending pressure
MVEI: Mechanical ventilation through endotracheal intubation
VAP: Ventilator-associated pneumonia
BPD: Bronchopulmonary dysplasia
ES: Extubation success
EF: Extubation failure
NICU: Neonatal intensive care unit
GA: Gestational age
BW: Birth weight
SGA: Small for gestational age
PCO_2_: Partial pressure of carbon dioxide
EOS: Early onset sepsis
HFV: High-frequency ventilation
CMV: Conventional mechanical ventilation
HB: Hemoglobin concentration
ALB: Serum albumin
GLB: Serum globulin
FiO_2_: Fraction of inspired oxygen
PMA: Postmenstrual age
NEC: Neonatal necrotizing enterocolitis
LOS: Late-onset sepsis
ROP: Retinopathy of prematurity
ROC: Receiver operator characteristic
C-index: Concordance index
CI: Confidence interval
IVH: Intraventricular hemorrhage
PDA: Patent ductus arteriosus.
